# Practical Success of Biomanipulation using Filter-Feeding Fish to Control Cyanobacteria Blooms: A Synthesis of Decades of Research and Application in a Subtropical Hypereutrophic Lake

**DOI:** 10.1100/tsw.2001.67

**Published:** 2001-08-08

**Authors:** Ping Xie, Jiankang Liu

**Affiliations:** Donghu Experimental Station of Lake Ecosystems, The State Key Laboratory for Freshwater Ecology and Biotechnology of China, Institute of Hydrobiology, Chinese Academy of Sciences, Wuhan 430072, China

**Keywords:** Cyanobacteria blooms, filter-feeding fish, biomanipulation, subtropical lake, silver carp, bighead carp, Microcystis, zooplankton

## Abstract

337 Lake Donghu is a 32-km 2 shallow, subtropical lake near the Yangtze River (P.R. China) that has experienced dramatic changes in the past five decades. These changes include: (1) a trophic state change from mesotrophy to hypertrophy; (2) dense blooms of cyanobacteria during every summer from the 1970s to 1984; (3) a cessation of blooms starting in 1985, with no recurrence; and (4) an increase, coincident with bloom declines, in the production of silver and bighead carp (filter-feeders) by more than tenfold. There are several possible explanations for the disappearance of blooms, including changes in nutrient concentrations, increased zooplankton grazing, and increased grazing on algae by fish. The long-term data suggest that changes in nutrients or in zooplankton were not important, but that the remarkably increased fish densities might have played the key role. To test this hypothesis, in situ enclosure experiments were conducted in three years. The main conclusions are as follows: (1) an increased stocking of the lake with carp played a decisive role in the elimination of cyanobacteria blooms; (2) both silver and bighead carp can eliminate cyanobacteria blooms directly by grazing; (3) zooplankton cannot suppress the blooms; and (4) the lake still is vulnerable to the outbreak of blooms, should fish grazing decline. The critical biomass of carp is approximately 50 g m 3 . The results suggest the applicability of a new food-web manipulation (increased stocking with filter-feeding fish) for controlling cyanobacteria blooms in hypereutrophic lakes. The approach differs from traditional biomanipulation in Europe and North America, where piscivores are added to control planktivores, and this in turn increases zooplankton and decreases algae. The new biomanipulation method is being used or being tested to counteract cyanobacteria blooms in many Chinese lakes such as Lake Dianchi?Xie and Liu: Biomanipulation to Control Cyanobacteria TheScientificWorld (2001) 1, 337-356 in Yunnan Province, Lake Chaohu in Anhui Province, and Lake Taihu in Jiangsu Province. The method has great potential as an important component of an integrated approach to counteract cyanobacteria blooms, especially in lakes where nutrient inputs cannot be reduced sufficiently, and where zooplankton cannot effectively control phytoplankton production.

## GENERAL FEATURES OF THE LAKE DONGHU ECOSYSTEM

Lake Donghu (30°33´N, 114°23´E) (Fig. 1) is located in Wuhan City, the capital of Hubei Province. It has a total surface area of 32 $km^2$ and a surface that is ~21 m above sea level. The outlet of the lake joins the Yangtze River through the Qingshan Canal and is approximately 5 km from the river. The mean and maximum water depths are 2.2 and 4.8 m, respectively. Hydraulic residence time of the lake is ~0.4 y. The catchment area is ~97 $km^2$.

In the latter half of the 1960s, Lake Donghu was divided into several sections by artificial dikes; Guozhengu Hu, Tanglin Hu, Hou Hu, and Niuchao Hu are the major lake sections. Since there remain only minimal interconnections, these sections are effectively isolated from each other. The Guozheng Hu (the site of sampling Stations I and II) is the most heavily impacted by anthropogenic nutrient inputs

Lake Donghu has multiple uses to society, including water supply, recreation, and commercial fishing. The lake is intensively used for fish production; stocking of the planktophagous silver and bighead carp increased the fish production more than tenfold in recent decades. The northwestern shoreline has a park and recreation area for the citizens of Wuhan, with museums, a botanical garden, observation towers, restaurants, swimming sites, and sight-seeing boats. There are approximately 100 factories (including a large steel plant) around the lake.

Dissolved oxygen concentrations in the lake water are often high. Super-saturation frequently occurs in the warm season, and nowhere is there an oxygen-free layer that might be insufficient for aquatic animals. The minimum monthly temperature (usually in January) varies from 2.6° to 4.6°C, and the maximum monthly temperature (usually in July) varies from 28.8° to 31.4°C[1]. Vascular aquatic plants are scarce in the lake, and their contribution to total system primary production is negligible[2]. Phytoplankton production, on the other hand, is very high. The dominant fish are the filter-feeding silver carp (*Hypophthalmichthys molitrix*) and bighead carp (*Aristichthys nobilis*). There also are some large piscivorous fish, but they occur at much lower densities. Research has indicated that fish grazing might significantly impact the lake's plankton[3].

## THE RISE AND FALL OF CYANOBACTERIA BLOOMS IN LAKE DONGHU

The annual average gross productivity of phytoplankton displayed a steady increase from the 1960s to early 1980s and then remained relatively stable (Fig. 2). The composition of the algal community also displayed marked changes in the 1950s and 1970s. In terms of individual numbers, in 1956 to 1957 Pyrrophyta ranked the first and Bacillariophyta were second; the two

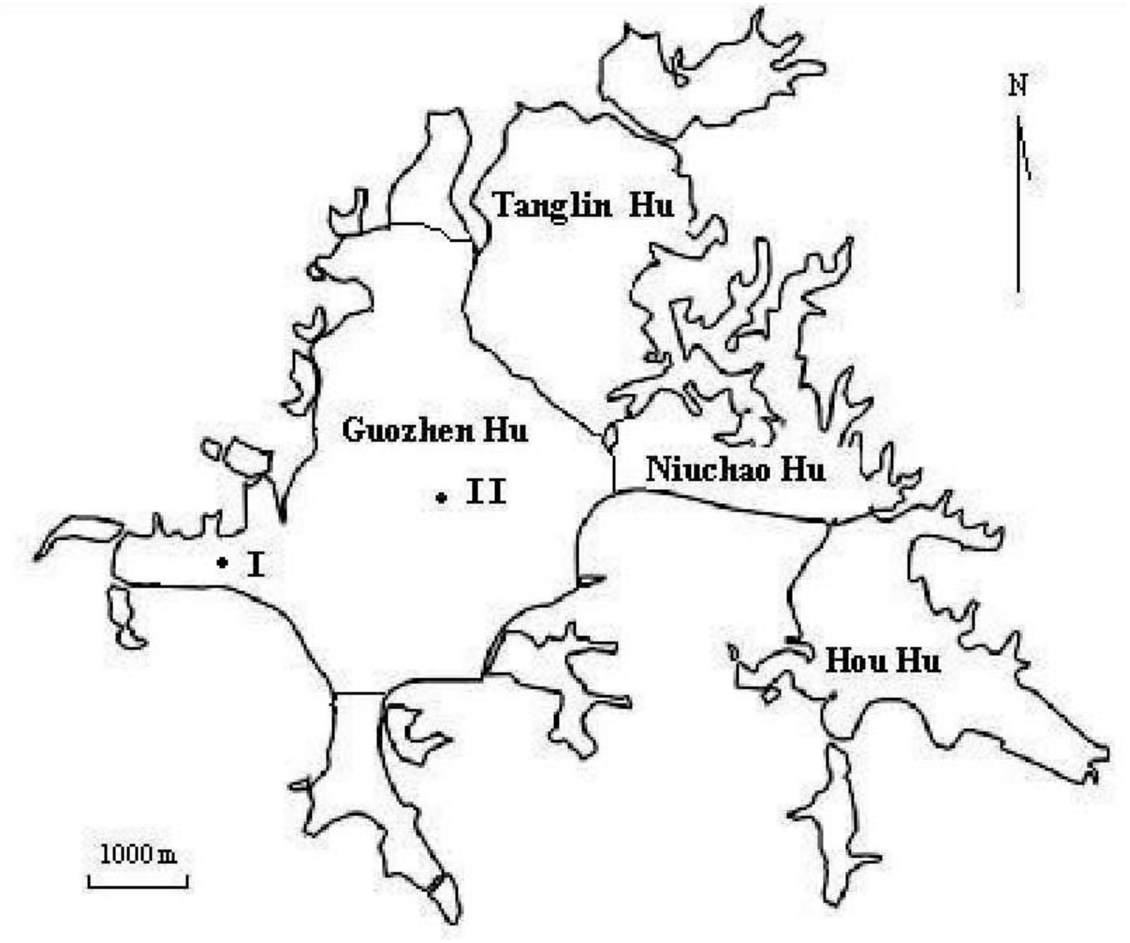

**FIGURE 1.** Map of Lake Donghu, P.R. China, showing major sections of the artificially divided lake.

phyla constituted 60 to 70% of total algal density. Cyanophyta and Chlorophyta were far less abundant. After the 1960s, Cyanophyta and Chlorophyta increased in density and accounted for >50% of the total algae[5]. No data were available to estimate biomass of phytoplankton before 1979 (i.e., cells or colonies were counted, but not measured). However, we suspect that the relative biomass of Cyanophyta also increased dramatically because the taxa that became dominant (including *Anabaena*, *Aphanizomenon*, and *Microcystis*) occur as large colonies[1].

The most visible sign of cultural eutrophication in Lake Donghu was the outbreak of cyanobacteria blooms, with unsightly and odorous scum on the water surface every summer from the 1970s until 1984 (Fig.3). In 1985 blooms did not occur for the first time, and they have not re-appeared in the last 16 years. A comparison of the average proportion of annual biomass of dominant phytoplankton at two sampling stations of Lake Donghu in 1979–1982 vs. 1989–1992 indicates this change (Fig. 4). During 1979–1982, when heavy cyanobacteria blooms occurred in the summer of each year, colony-forming *Microcystis* and filamentous *Anabaena* and *Oscillatoria* dominated the phytoplankton. During 1989–1992, the dominant phytoplankton taxa were *Cyclotella* (a centric diatom) and *Cryptomonas* (a cryptomonad). Cyanobacteria were mainly comprised of *Oscillatoria* and *Merismopedia*[3]. These cyanobacteria do not generally form noxious blooms.

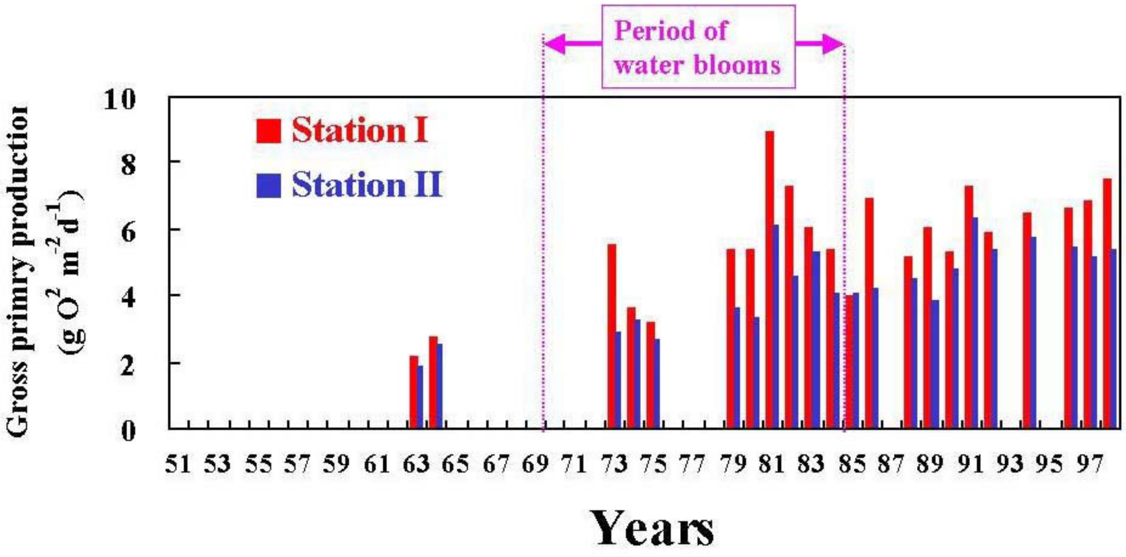

**FIGURE 2.** Annual mean gross primary production at Stations I and II of Lake Donghu (adapted from Xie et al.[4]).

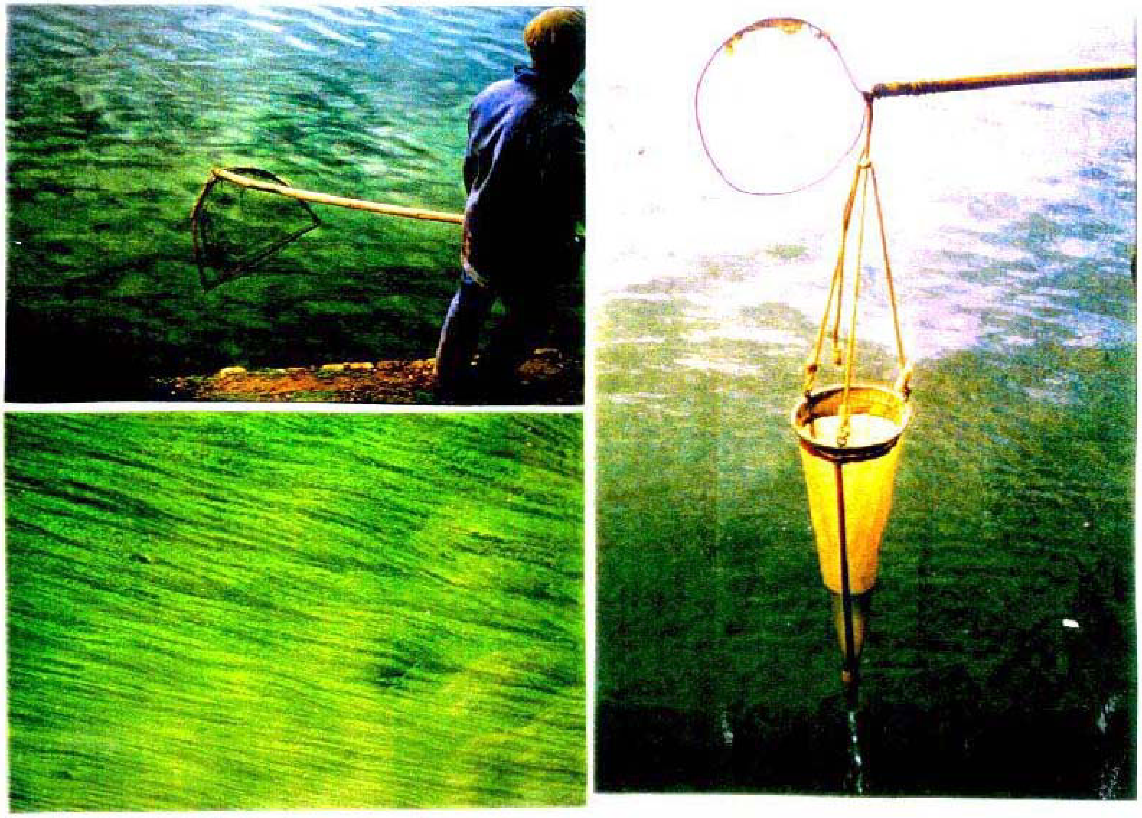

**FIGURE 3.** Cyanobacteria blooms in Lake Donghu on October 9, 1981 (photo by K. Tatsukawa).

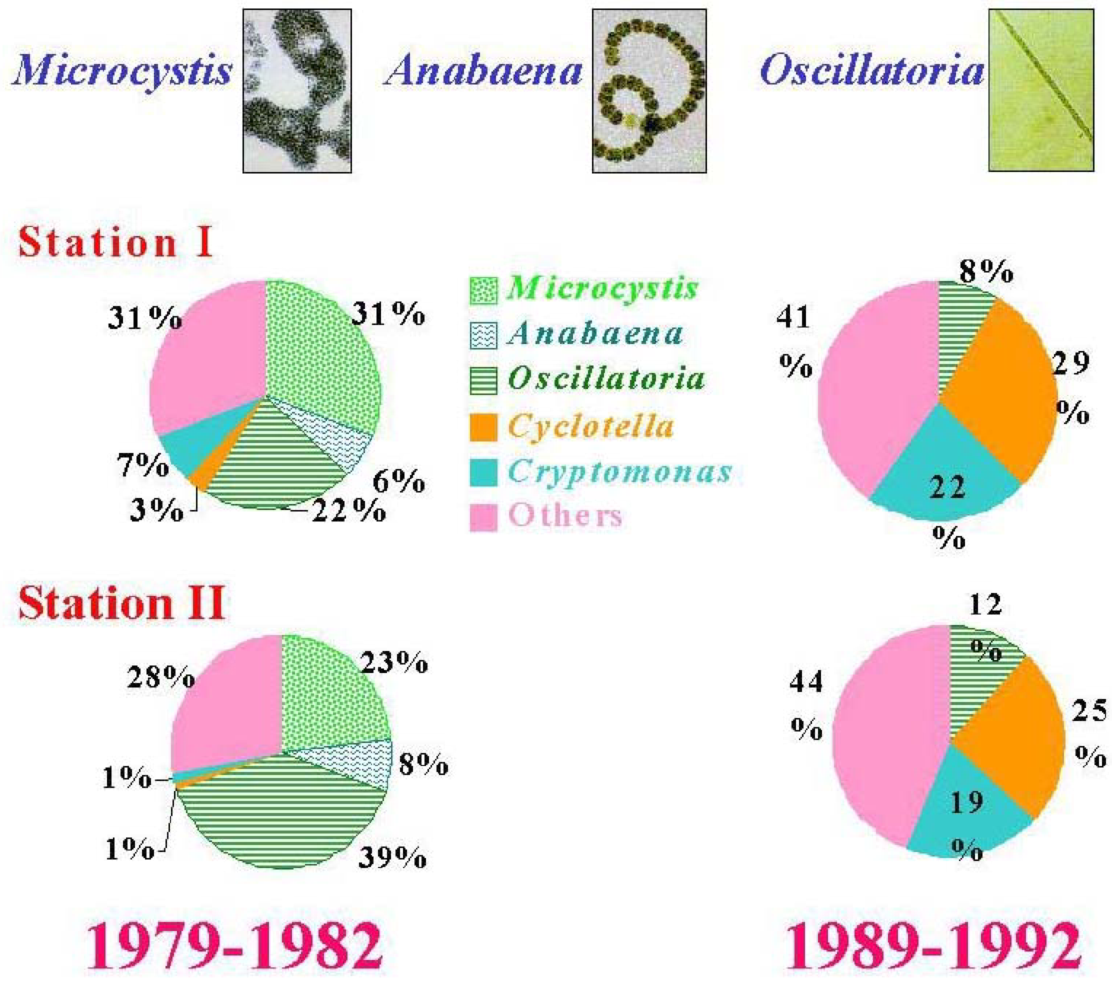

**FIGURE 4.** Annual mean biomass (%) of dominant phytoplankton at two sampling stations of Lake Donghu between 1979–1982 and 1989–1992 (1979–1982 data from Wang[6]).

## HISTORICAL CHANGES IN NUTRIENTS, ZOOPLANKTON, AND FISH

### Nutrients

Cultural eutrophication has occurred over the past decades in Lake Donghu[5,7,8]. Water samples collected at the mid-lake Station II indicate that ammonia nitrogen increased from 0.043 mg/l in 1957 to 0.361 mg/l in 1998. The mass stocking of fish has been suggested as a possible cause. However, neither feed nor fertilizer is applied to the lake for the stocked fish; the stocked fingerlings feed just like the wild population, solely on the food organisms naturally present, and then are removed from the water annually[1]. Hence, there probably is a net removal of nutrients from the lake by fish. In contrast, with the ever-increasing human population and rapid development of industry, agriculture, and animal husbandry in the drainage basin, larger amounts of nitrogen and phosphorous have been entering the lake each year in the form of semi-treated effluents[5,7].

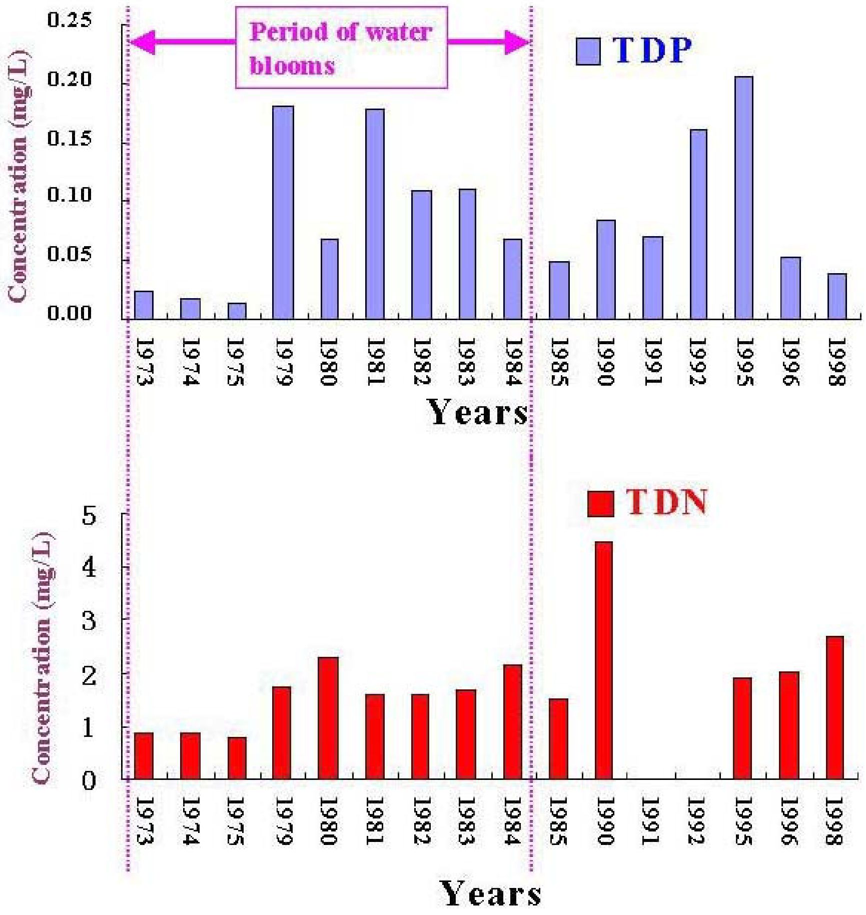

**FIGURE 5.** Annual mean concentrations of total dissolved nitrogen (TDN) and total dissolved phosphorus (TDP) at mid-lake Station II of Lake Donghu during 1973–1998 (adapted from Tang and Xie[7]).

During the 1970s and 1990s, the concentrations of TDN and TDP showed great variation but no obvious downward or upward trends (Fig. 5) that might explain the changes in phytoplankton observed in that time period. Likewise, the ration of TDN to TDP did not systematically vary.

## Zooplankton

Densities of protozoa, rotifers, copepods (including nauplii), and cladocerans were evaluated from 1956 to 1996 (Fig. 6). Although protozoan densities varied from year to year, they did not systematically increase or decrease. Densities of rotifers, cladocerans, and copepods all peaked in the early 1980s, and then declined greatly. The copepods were mainly comprised of predaceous cyclopoids, while the herbivorous calanoids are relatively low in numbers. Before 1987, two large-bodied species, *Daphnia galeata* and *Daphnia carinata*, dominated the cladoceran community. Later, these species were replaced by the smaller *Moina micrura*. The population of *Diaphanosoma brachyurum*, an intermediate-sized cladoceran, remained rather stable[4]. Because declines in large cladocerans occurred in approximately the same time frame as the disappearance of cyanobacteria blooms, one cannot attribute the loss of blooms to increased zooplankton grazing. Rather, one might presume that some common factor led to the zooplankton changes and bloom demise.

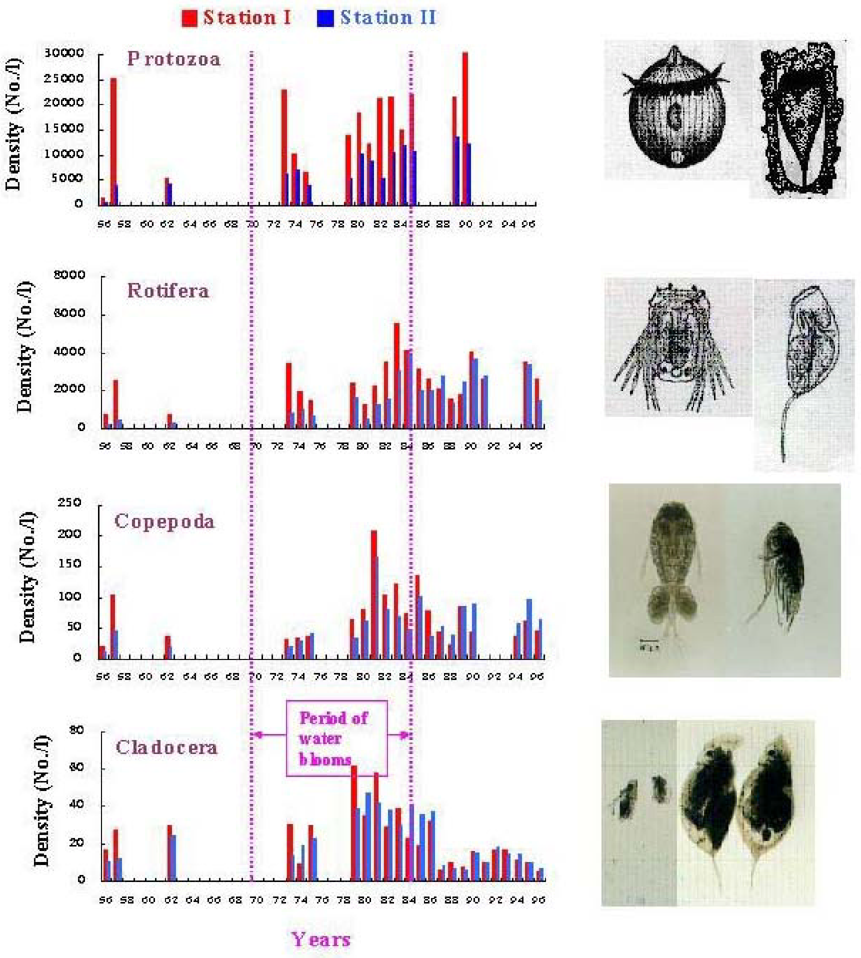

**FIGURE 6.** Annual mean densities of total Protozoa, Rotifera, Copepoda (including nauplii), and Cladocera at Stations I and II of Lake Donghu (adapted from Xie and Yang[9]).

## Fish

Commercially harvested fish constitute the major part of the fishery in Lake Donghu[1]. The state-owned Donghu Lake Fish Farm was established in 1951, and since 1971 it has stocked the lake every year with mainly planktivorous filter-feeding silver and bighead carp. As indicated, the carp are not provided with fertilizer or other food sources, but rather, they feed on natural food items available to them in the lake water. In the 1950s and 1960s, the annual fish yield of Lake Donghu varied from 39 to 178 kg/ha, with a mean of 92 kg/ha (Fig. 7). During 1972–1978, a series of measures were taken to increase the fish yield of the lake. These included increasing the stocking density and the proportion of large-sized (>13 cm) fingerlings (Fig.7), reconstructing the fish screens, controlling the predatory fish[10], and adopting the method of bulk harvesting (Fig. 8). Of the total stocked fingerlings during the periods 1973–1978 and 1983–1997, silver carp constituted 46.5% (range 28.4–73.4%), bighead carp constituted 40.3% (range 18.4–63.2%), and a smaller percentage was of other fish. The annual fish yield increased steadily from 124 kg/h in 1971 to 1,068 kg/ha in 1997 (Fig. 7). From the 1970s on, more than 85% of the total fish yield was comprised of stocked species, especially in recent years, when >90% was from silver and bighead carp[11]. Taking into account the annual fish yield, the exploitation rate by the fishmen[12], and the daily ration of the fish[13], the estimated fish biomass in the lake is several times higher than that of the total plankton. Grazing pressure by the planktivorous fish should be rather strong in the lake[3,4,9].

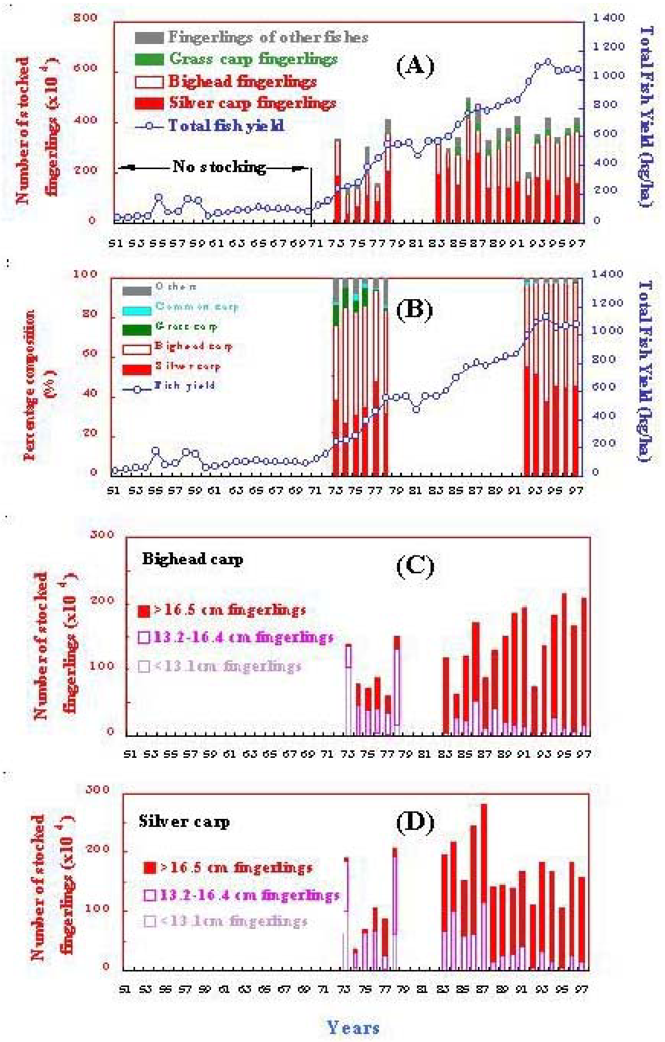

**FIGURE 7.** Annual fish yield and the number of stocked fingerlings (A), the percentage composition of the fish yield (B), the number of the stocked silver carp fingerlings of different sizes (C), and the number of the stocked bighead fingerlings of different sizes (D) (adapted from Xie et al.[4]). Stocking with fish-fry before the 1970s resulted in poor fish yield.

## EXPERIMENTAL STUDIES

To clarify the possible role of silver and bighead carp in the disappearance of cyanobacteria blooms from the lake since 1985, *in situ* enclosure (2.5 × 2.5 × 2 m) experiments were conducted in 1989, 1990, and 1992. The detailed results have been published elsewhere[14,15,16,17], so a general summary is provided here.

### First Experiment — 1989

This experiment was performed in order to determine (1) how the stocking of silver and bighead carp influences the community structure of phytoplankton, and (2) if dense stocking of these fish could eliminate a cyanobacterial bloom. Eight 2.5 × 2.5 × 2 m deep enclosures were set up in the Shuiguo basin of the Guozheng Hu section of the lake in May 1989. Only three of the eight enclosures could be used because strong winds destroyed the others. The enclosures of polyethylene sheets were open to air above and in direct contact with sediment underneath. The volume of the water in each enclosure was about 12.5 m$^3$.

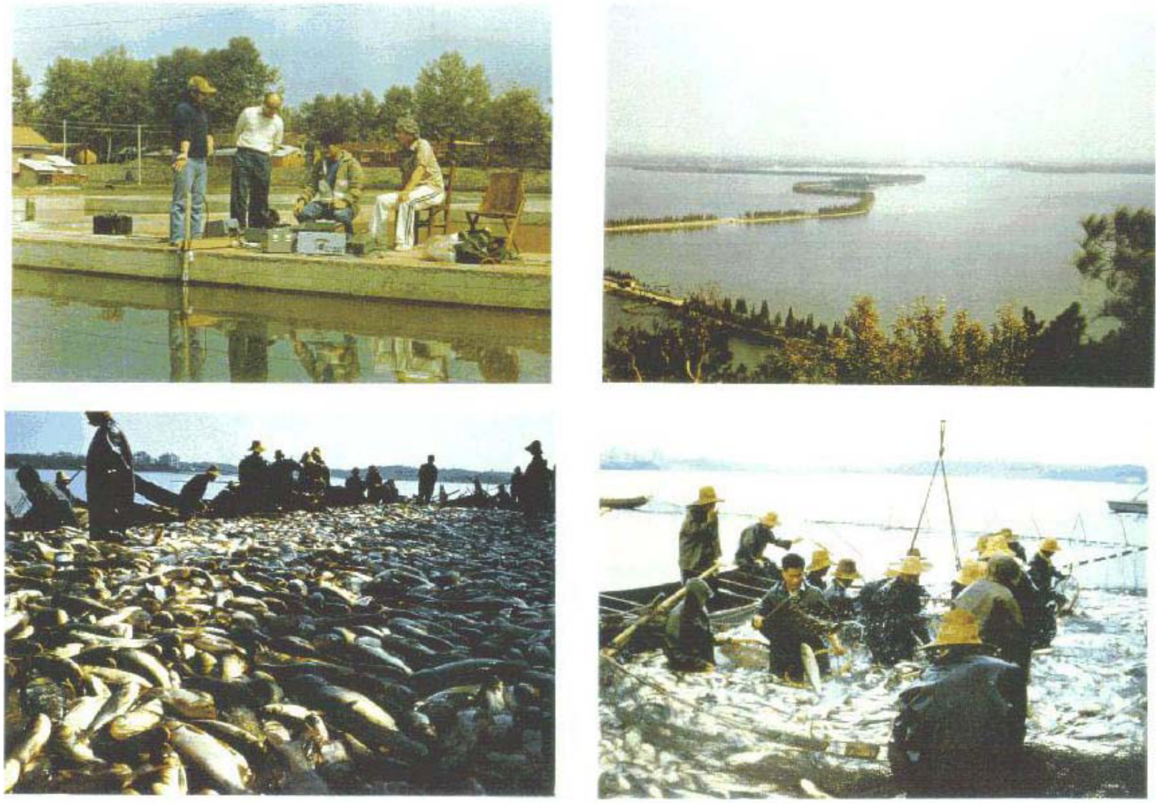

**FIGURE 8.** The harvested silver carp and bighead (lower left), the bulk harvesting by the fishermen (lower right), testing of echo-sounder ashore a fish pond (upper left) by scientists to estimate fish population of the lake, and the artificial dikes across Lake Donghu (upper right) (photos by K. Tatsukawa).

After initial sampling on May 17, six fish of both species were introduced into enclosure 3, twelve bighead carp into enclosure 5, and no fish were added to enclosure 7. The average individual weights at stocking were 75 g for silver carp and 380 g for bighead carp. The introduction of fish resulted in dramatic changes, not only in the total algal volume but also in the dominance of certain taxa in the phytoplankton community (Fig. 9). In terms of average algal volume, the dominant phytoplankton in the fish-stocked enclosures and the lake were quite similar, i.e., Cryptophyceae (mainly *Cryptomonas*) ranked first, Bacillariophyta (mainly *Cyclotella*) second, and Chorophyceae third. However, the situation in the fish-free enclosure 7 was quite different, where a bloom of *Microcystis* appeared after mid-June and persisted throughout the experiment. Despite the lack of replication, the dramatic differences in treatments and similar responses of the experimental units to what had been observed historically in the lake supported the hypothesis of fish control of phytoplankton blooms.

## Second Experiment — 1990

After the finding of a cyanobacteria bloom in the fish-free enclosure in 1989, a second experiment was conducted in order to (1) induce water blooms in more enclosures, (2) introduce silver and bighead carp to the enclosures with blooms to determine if the fish could graze down the algae, and (3) monitor changes in the size structure of the phytoplankton biomass.

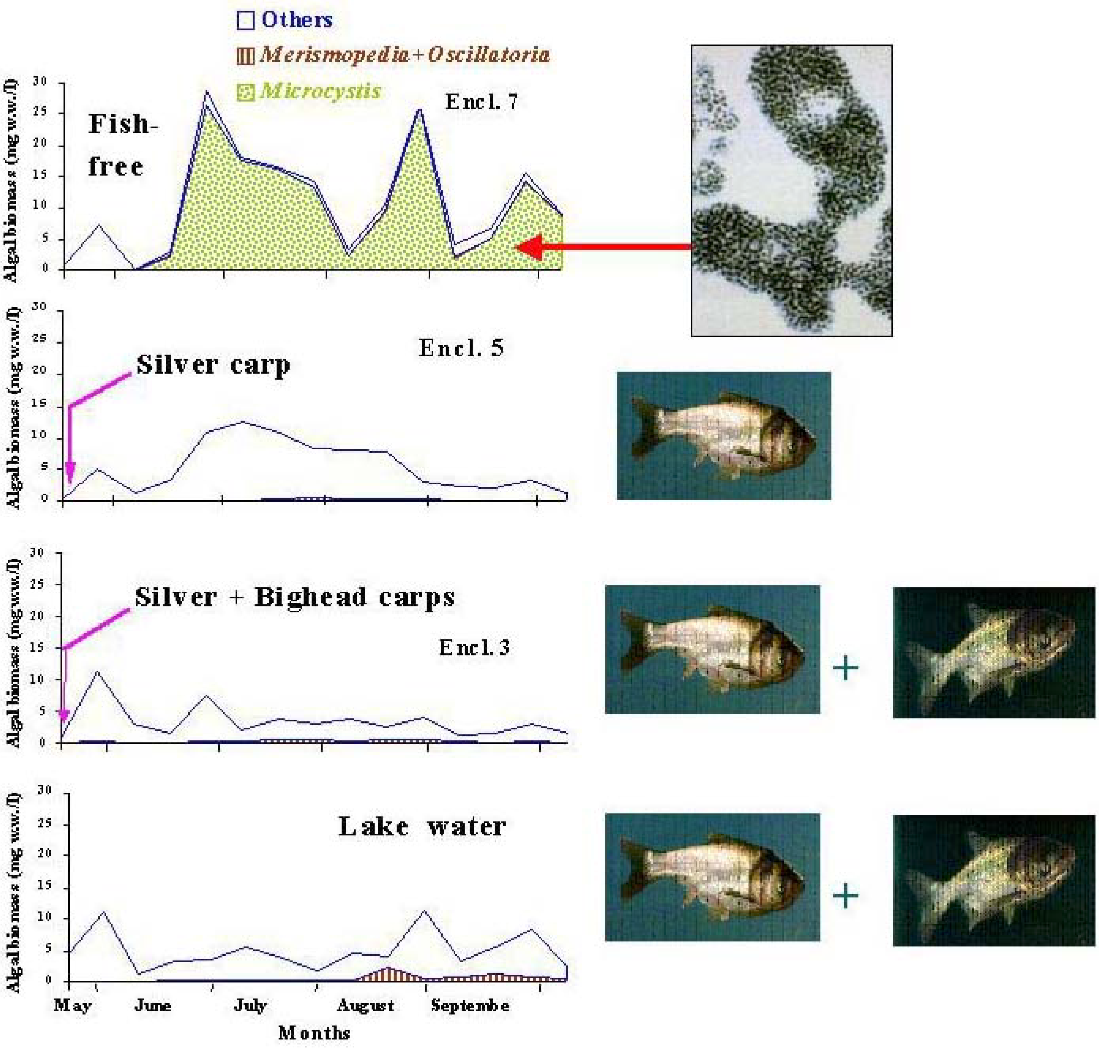

**FIGURE 9.** Changes in the biomass of *Microcystis* and other phytoplankton in the enclosures and the surrounding lake water in Lake Donghu during May-October, 1989 (adapted from Xie and Liu[14]).

Six enclosures were used with the same location and design as in the previous year. Initial sampling was done on April 5. On the next day, four silver carp and two bighead carp were introduced into enclosures 3 (average weight 21g/ind. for silver carp and 32 g/ind. for bighead) and 4 (average weight 23 g/ind. for silver carp and 32 g/ind. for bighead). The other enclosures were not stocked with fish. There were pronounced phytoplankton responses to the treatments, both in terms of total biomass (measured as chlorophyll *a*) and biomass of small phytoplankton (measured as chlorophyll *a* in 30 µm filtered water) (Fig. 10). In the enclosures stocked with fish (3 and 4), total chlorophyll *a* remained low throughout the experiment, and, on the average, more than 80% of the biomass was attributed to small taxa. During April and May the enclosures with fish displayed a similar situation in terms of chlorophyll *a*. However, dramatic changes followed. A bloom was observed in enclosure 2 in mid-June, in enclosures 1 and 5 in late-June, and in enclosure 6 in mid-July. With the appearance of blooms in these fish-free enclosures, the dominant phytoplankton shifted to *Microcystis*. These results confirmed the findings of the 1989 study. On July 20, after water samples were taken for phytoplankton analysis, six silver carp

(average weight 148 g/ind.) were introduced into enclosure 1, six bighead carp (average weight 176 g/ind.) into enclosure 2, and three silver carp (average weight of 152 g/ind.) plus three bighead carp (average weight 172 g/ind.) into enclosure 5. No fish were stocked into enclosure 6 throughout the experiment. The introduction of fish caused drastic declines in the total biomass of phytoplankton in enclosures 1, 2, and 5. In enclosure 1, the cyanobacteria bloom disappeared within 10 days, and in enclosures 2 and 5, blooms disappeared 18 days later. In all cases the dominant phytoplankton shifted to <30 µm taxa. In the fish-free enclosure (6), the cyanobacteria bloom persisted throughout the experiment.

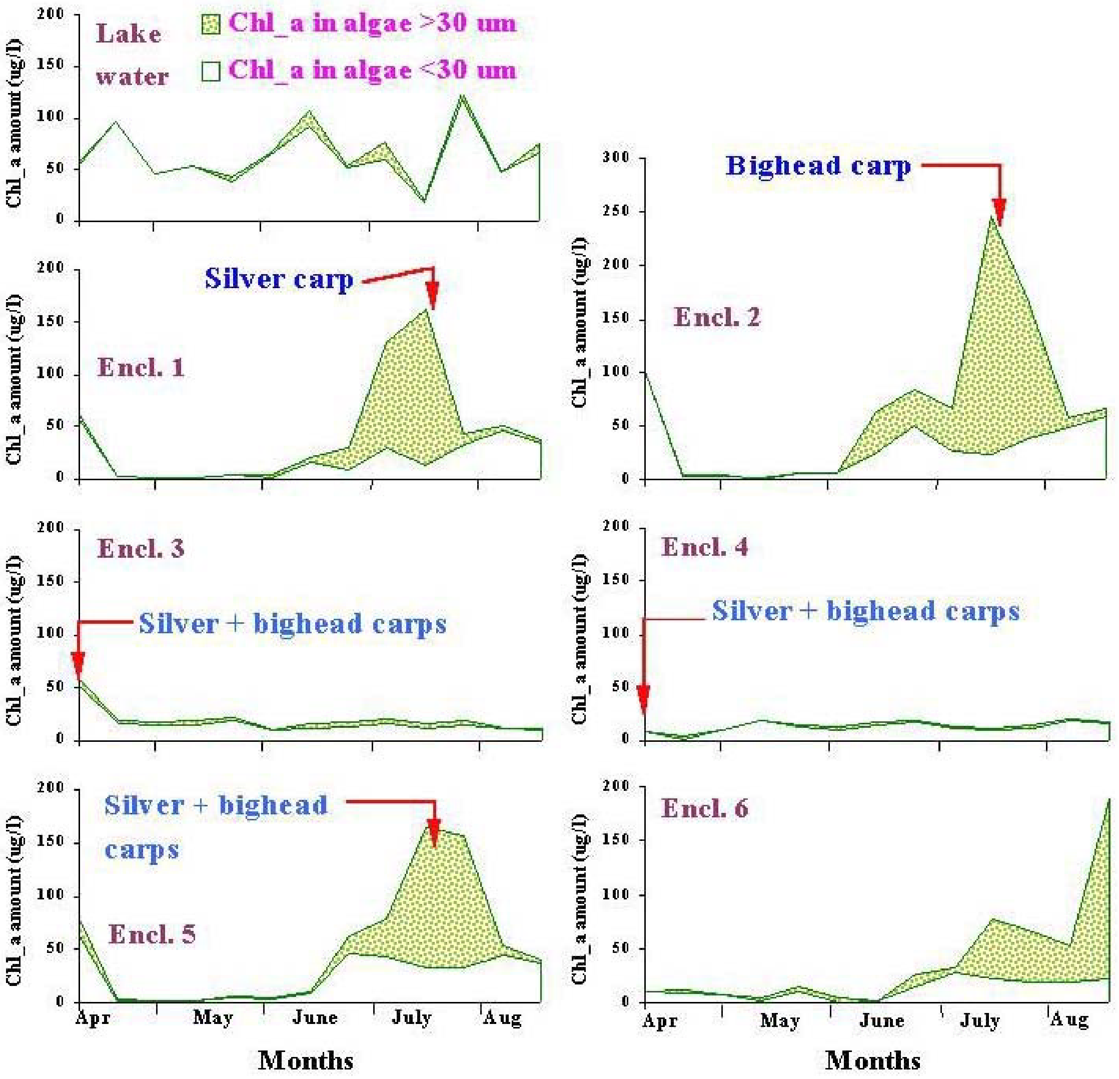

**FIGURE 10.** Changes in chlorophyll *a* amount in both total phytoplankton and those <30 µm phytoplankton in enclosures and the surrounding lake water in Lake Donghu during April-August, 1990 (adapted from Xie and Liu[15]).

## Third Experiment — 1992

A third experiment addressed the question of whether a different fish (grass carp—*Ctenopharyngodon idellus*) could have similar effects on phytoplankton blooms. In this case the treatments included: (1) two planktivorous carp at different densities stocked into enclosures with resurgent blooms; (2) grass carp stocked into enclosures with blooms; and (3) monitoring of responses in terms of phytoplankton biomass and size, as above. A total of eight enclosures were used (two per treatment, plus controls) at the same location as in previous years. The experiment was carried out during July–September. The lake water was isolated in the enclosures on July 6, and the first sampling was made on July 7. At the beginning of the experiment, no blooms occurred. Two weeks later, a thin water bloom of mainly green algae appeared in most of the enclosures. Subsequently, the dominant taxa changed to cyanobacteria, mainly *Microcystis*. By the end of July, a dense *Microcystis* bloom occurred in most enclosures, and by mid-August, all enclosures had this condition. As noted previously, the open lake did not support cyanobacteria blooms at this time in its history. On August 21, three grass carp were stocked into enclosure 3 (average weight 187 g/ind.) and 4 (average weight 229 g/ind.), three silver carp into enclosure 5 (average weight 137 g/ind.), two silver carp (average weight 133 g/ind.) into enclosure 6, and four bighead carp into both enclosures 7 (average weight 233 g/ind.) and 8 (average weight 216 g/ind.). No fish were stocked into enclosures 1 or 2. The introduction of bighead carp to enclosures 7 and 8 caused dramatic declines in total phytoplankton biomass (Figs. 11, 12), and about two weeks later, the cyanobacteria blooms disappeared. The introduction of silver carp to enclosures 5 and 6 also decreased phytoplankton biomass, but there was still a bloom in these enclosures at the end of the experiment. The introduction of grass carp to enclosures 3 and 4 did not reduce phytoplankton biomass, and dense blooms persisted until the end of the experiment. In the fish-free enclosures, heavy blooms also persisted. Changes in the size structure of phytoplankton were variable among the enclosures. In the fish-free enclosures and the enclosures stocked with grass carp, phytoplankton biomass was dominated by the larger (>30 μm) size class. In enclosures stocked with the planktivorous fish (silver and bighead carp), phytoplankton biomass was dominated by the smaller (<30 μm) fraction, especially at higher fish densities. There have been contradictory reports in the literature on the impact of silver carp on phytoplankton. Through enclosure experiments in a small Polish lake (Lake Warniak), Kajak et al.[18] found that silver carp reduced blue-green algae and total phytoplankton biomass while increasing the proportion of dinoflagellates and nano-plankton. However, it also was reported that silver carp stimulated phytoplankton and might be ineffective for the control of algal blooms[19,20,21]. Our results provide strong support for the view that the latter conclusion is not always valid and that, in fact, silver and bighead carp are highly effective in controlling noxious blooms of algae.

## Summary of Experimental Results

Based on the results of the three *in situ* enclosure experiments, the following conclusions are reached: (1) the increased stocking of the filter-feeding silver and bighead carp played a decisive role in the elimination of water bloom from Lake Donghu after 1985; (2) the lake remains vulnerable to the outbreak of *Microcystis* blooms; and (3) both silver and bighead carp can eliminate cyanobacteria blooms directly by grazing. Our results also indicate that zooplankton cannot suppress the blooms in this lake. When the experimental results are integrated with echo-sounding records of fish biomass and the fishery production of the lake over the years, it is concluded that the recurrence of blooms can be prevented if the biomass of silver plus bighead carp is held at or above ~50 $g/m^3$.

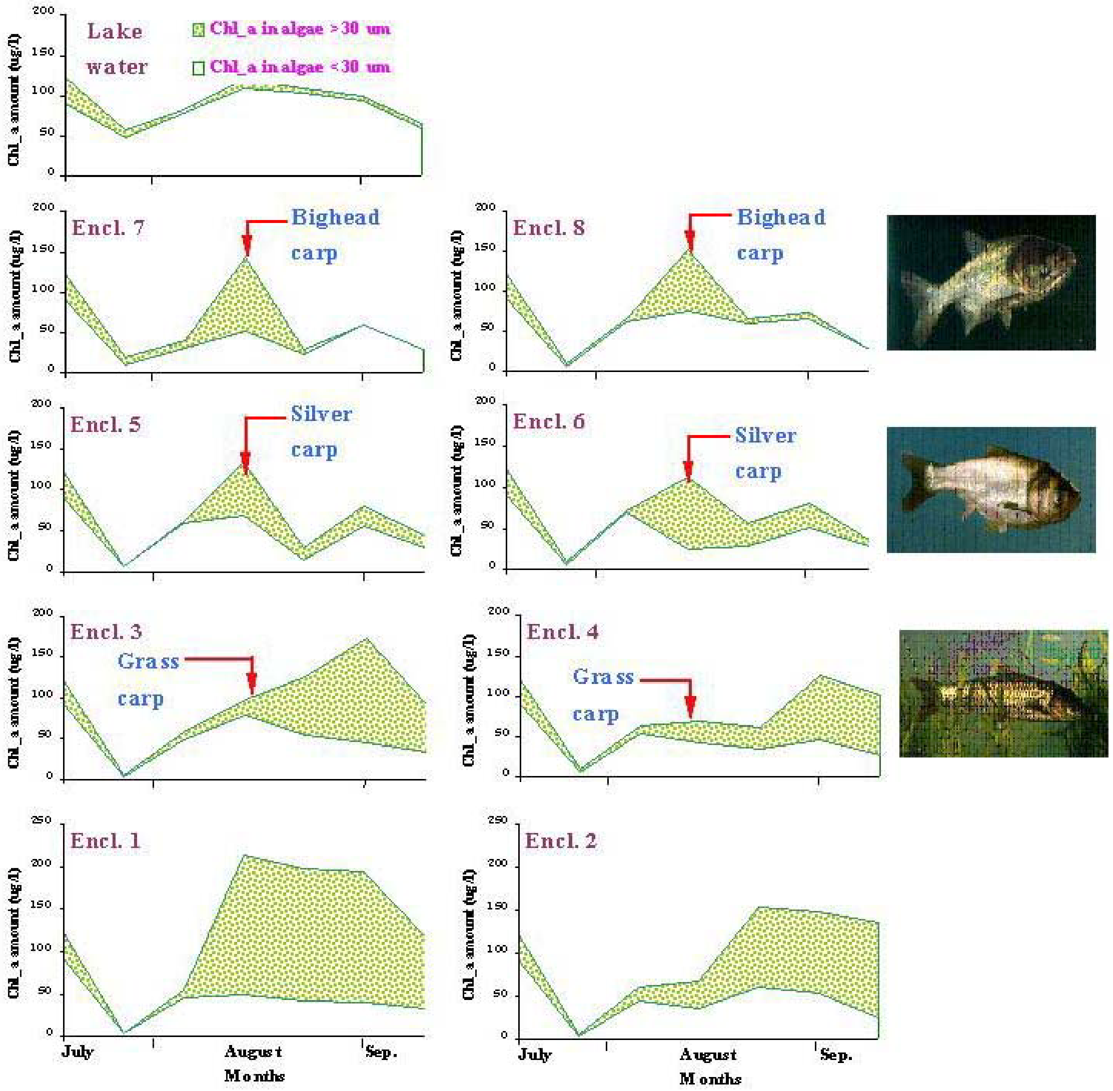

**FIGURE 11.** Changes in chlorophyll *a* amount in both total phytoplankton and those <30 µm phytoplankton in enclosures and the surrounding lake water in Lake Donghu during July–September, 1992 (adapted from Xie et al.[16]).

## DIGESTION OF CYANOBACTERIA BY SILVER AND BIGHEAD CARP

Another issue that has been a subject of debate is the digestibility of cyanobacteria grazed by silver and bighead carp. Examinations of gut contents from silver carp in a culture pond indicated that most of the algae present appeared intact in both the fore- and hind-gut[22,23,24]. A prerequisite to the utilization of the cell contents is breakdown of the algal cell wall by one of three mechanisms, i.e., acid hydrolysis, enzymatic digestion, or mechanical trituration[25]. In filter-feeding fish with no stomachs, such as silver carp, the pH of the gut fluids usually is >6. No cellulase in the gut of silver carp was found[26], and *in vitro* incubation of a filamentous cyanobacterium (*Anabaena flos-aquae*) with digestive juices of silver carp resulted in only 10% of the algal protein being hydrolyzed to free amino acids[24]. It was concluded that silver carp are omnivorous rather than herbivorous[25,26].

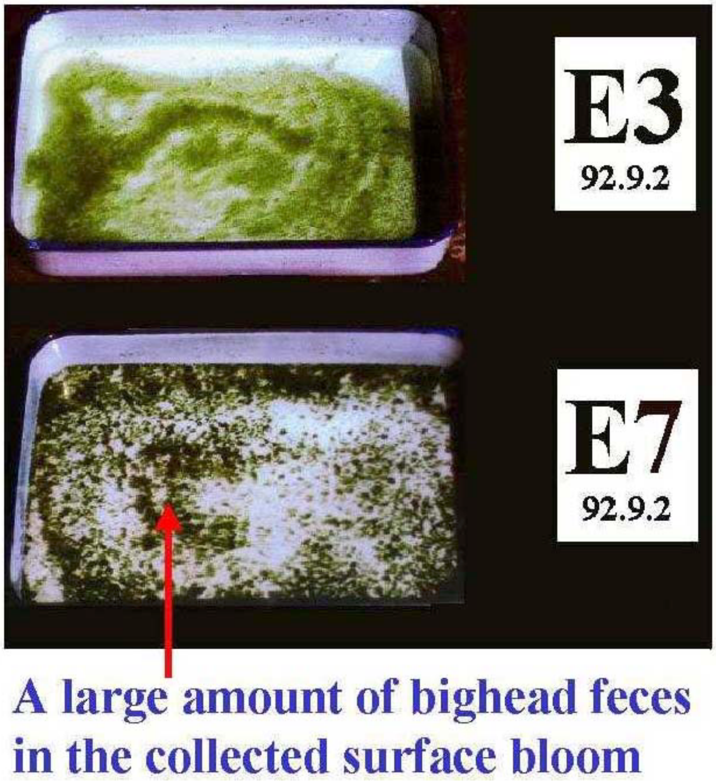

**FIGURE 12.** *Microcystis* blooms collected from the surface of enclosures 3 and 7 on September 2. On August 21, 932 g of bighead carp was stocked into enclosure 7, while 561 g of grass carp was stocked into enclosure 3.

Xie[27] examined the process and digestion of the centric diatom *Cyclotella* in the alimentary canal of silver carp. The results indicate that 67% of the cells was damaged as a result of ingestion and digestion by silver carp, of which as much as 52% occurred in the esophagus while only 15% could be attributed to digestion in the intestine. In other words, mechanical crushing of a sufficient proportion of ingested algal cells occurred apparently as a result of grinding by the pharyngeal teeth in the esophagus. Similar results were obtained for bighead carp[28].

On the other hand, studies using radioisotope techniques indicate that silver carp digests green algae and cyanobacteria efficiently. Using cultured *Anabaena flos-aquae* labeled with $^{14}$C, silver carp fry ingested 10–13% of their body weight per day, and 17% of the ingested algae was assimilated[29]. Using cultured, $^{14}$C-labeled green algae as food, silver carp were found to assimilate 42% of *Closterium* and 14% of *Selenastrum*[30]. A series of tracer experiments using *Anabaena spiroides* collected from a fish pond and then labeled with $^{32}$P indicated that silver carp ingest algae at a rate of 12–22% of their body weight per day, and that 65% of the ingested material is assimilated[31]. Tracer experiments with $^{32}$P also indicate that silver carp assimilate 35–48% of ingested *Microcystis aeruginosa*, 17–36% of *Euglena*, and 44–60% of *Scenedesmus obliquus*[32,33]. Similarly, bighead carp assimilate 23–38% of ingested *Microcystis aeruginosa* and 25–53% of *Euglena* sp.[33].

Based on these results, the feeding mechanisms of silver carp are summarized as follows. The breakdown of cell walls of ingested algae mainly takes place in the esophagus by mechanical grinding by pharyngeal teeth[27]. Some algal cells remain intact, with the proportion varying among species. On passage through the intestine, few or only a very small proportion of the intact algae are destroyed, especially those algae with cellulose cell walls such as green algae, due to the lack of cellulase in the intestine. This explains the erroneous conclusion by some authors that stomachless fish do not use algae as a food resource[22,24,25,26]. Assimilation of the contents of broken algal cells takes place in the intestine even though the intact algal cells may change little in their proportion on passage through that organ, explaining the effective assimilation of silver carp on some cyanobacteria and green algae observed with isotope techniques. The presence of intact or mobile alga in the hind-gut or feces[22,23,24] does not necessarily mean that the species are indigestible, since Xie[27] shows that only one third of the ingested *Cyclotella* remained intact in the feces. The incomplete digestive mechanism on algae may reflect an adaptive strategy for stomachless filter-feeding fish that continuously feed on small, suspended particles including not only plankton but also large amounts of organic detritus of low nutritional value. A similar mechanism also occurs in bighead carp[28].

## POTENTIAL FOR USE OF SILVER AND BIGHEAD CARP TO CONTROL CYANOBACERIA BLOOMS

### Measures for the Control of Cyanobacterial Blooms

There are several commonly used measures to counteract cyanobacteria blooms in hypereutrophic lakes[34]. These include: (1) chemical algicides, especially copper sulfate; (2) nutrient input reduction and manipulation (e.g., N:P ratios); (3) vertical de-stratication through mechanical mixing or bubbling; (4) enhanced water flushing to reduce retention time; and (5) biological control. Option 1 is neither practical nor advised in larger ecosystems or any waters to be used for fishing, drinking, and other animal and human use, since algicides may have toxic effects to non-target species. Option 2 is the most commonly used and the method with the longest history in lake management. However, it is expensive and, in certain cases, where much of the input is from non-point sources, difficult to achieve in an effective manner. Some shallow lakes with high internal nutrient loading also do not respond for long time periods to external load reductions. Option 3 may be used in small, relatively shallow (i.e., <5 m) systems. Option 4 may be possible if abundant water supplies (i.e., upstream reservoirs) are available for flushing. Biological control (5) still is in an experimental stage but has been effectively applied to a number of lakes. It involves modifying the biological community in a manner that results in increased grazing pressure on the cyanobacteria. A common (traditional) biomanipulation approach is one where piscivorous fish are added to a lake in order to reduce the abundance of zooplankton-eating planktivores. This allows large-bodied zooplankton like *Daphnia* to increase, and with their increased grazing the biomass of phytoplankton is reduced. There is some controversy about the general applicability of this method of biomanipulation.

### Limitations of Traditional Food Web Biomanipulation

Limnologists traditionally considered pelagic communities to consist of components linked through a unidirectional flow of influence from nutrients → phytoplankton → zooplankton → fish[35]. In the 1970s, it was recognized that a significant reduction of zooplanktivorous fishes through enhancement of piscivorous fish could result in higher densities of zooplankton and consequently a substantial decline in algal biomass by zooplankton grazing[36,37]. Further experimental work by Shapiro et al.[38] led to subsequent investigations of this method of biomanipulation. This traditional approach (Fig. 13) may be effective in lakes that are not heavily enriched with nutrients, and with algae composed of small species. However, it may not be as applicable in hypereutrophic lakes, which tend to have large algae and sometimes only small zooplankton.

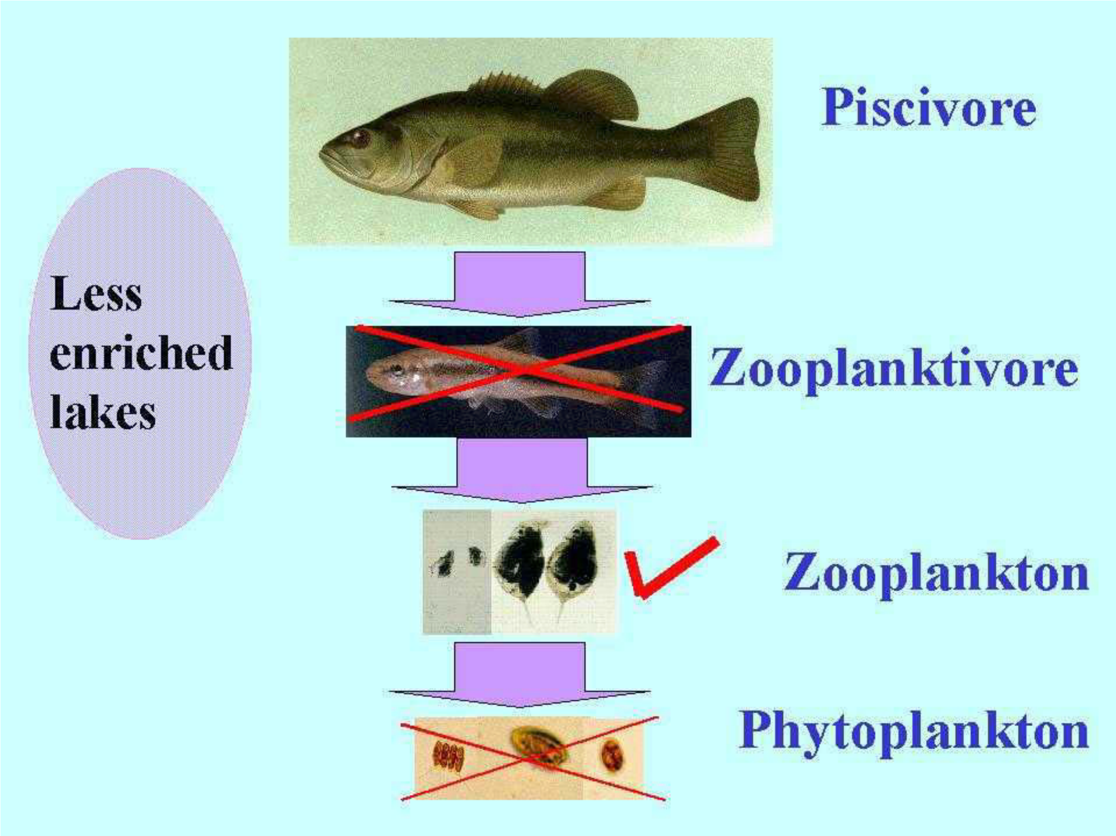

**FIGURE 13.** Conceptual diagram of traditional biomanipulation for the control of algal blooms.

The typical algal dominants may have (in addition to large size) defenses against grazers such as gelatinous or hard sheaths, and some produce toxins. It is reported that cyanobacteria blooms often are associated with a decline of zooplankton biomass and replacement of dominant zooplankton by smaller species of cladocerans, copepods, or rotifers[39,40,41,42,43]. It also is known that increased grazing pressure by zooplankton can shift the phytoplankton community toward large taxa, with little or no net change in total algal biomass[44,45,46].

## Advantages of Biomanipulation with Filter-feeding Fish

By manipulating the abundance of filter-feeding silver and bighead carp, we have been successful in eliminating heavy cyanobacteria blooms from hypereutrophic Lake Donghu for a period of 16 years. This practice disclosed the applicability of a new food-web manipulation, i.e., it is quite possible to eliminate the unsightly and odorous cyanobacterial blooms in hypereutrophic lakes by increasing the filter-feeding silver and bighead carp through stocking (Fig. 14). In contrast to particle-feeding fish that visually select individual prey items (primarily larger zooplankton)[47], filter-feeders collect food by passing water through their filtering apparatus, feeding on phytoplankton (usually >10 um), suspended detritus, and also zooplankton if they are unable to evade the fish. In this sense, silver and bighead carp are not only zooplanktovores but also algivores. This new method of biomanipulation is being used or tested to counteract cyanobacteria blooms in many Chinese lakes, including Lake Dianchi in Yunnan Province, Lake Chaohu in Anhui Province, and Lake Taihu in Jiangsu Province.

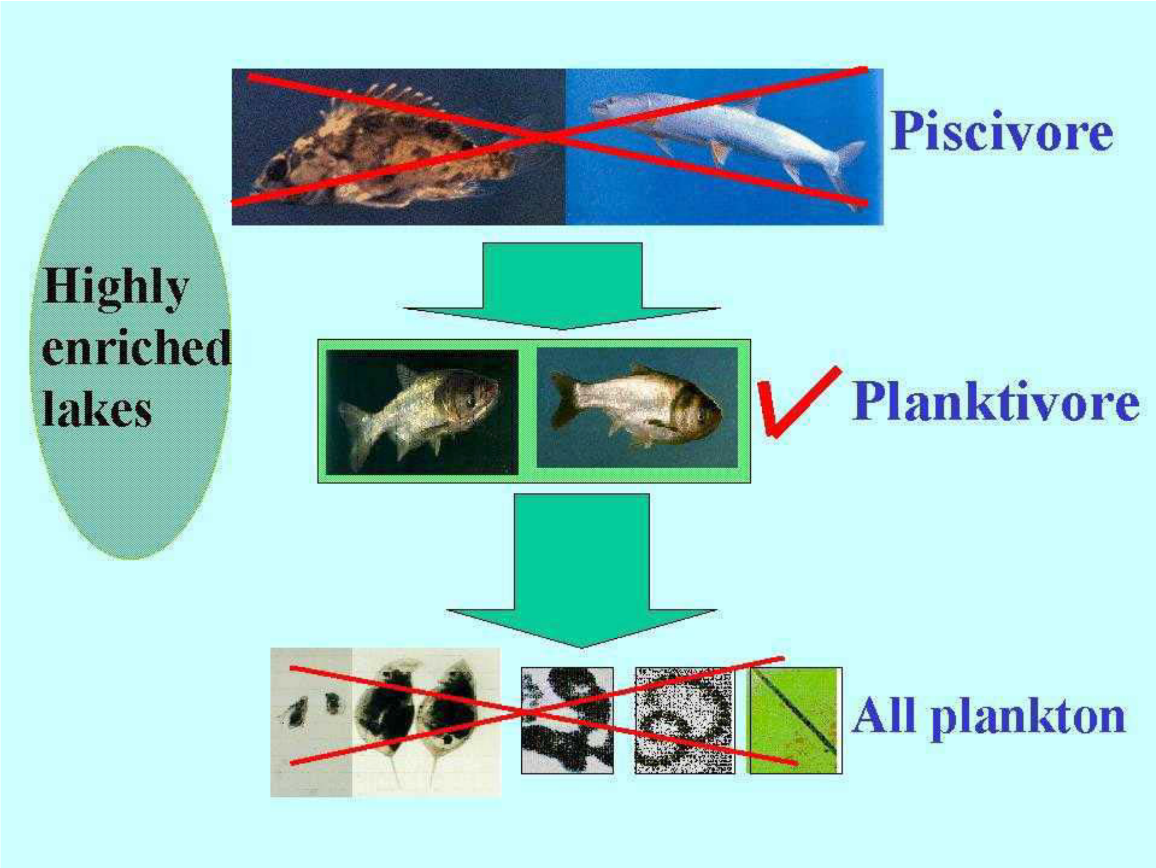

**FIGURE 14.** Conceptual diagram of the new biomanipulation by using filter-feeding silver and bighead carp to counteract cyanobacteria blooms.

Silver and bighead carp are native to eastern Asia[48]. Silver carp occur in rivers and lakes of China, North Vietnam, and Siberia. The natural range of bighead carp is smaller. This fish is found in China, from the Yellow River in the north to the Pearl River in the south. Silver and bighead carp contribute greatly to the world catch of freshwater fish (Fig. 15), and have been cultured for centuries in mixed cultures with other fish species, increasing total yield due to utilization of primary production. Since the 1960s, silver and bighead carp have also been introduced worldwide (Fig. 15). It is relatively easy to regulate the population size of the carp since they cannot reproduce naturally in lakes. In other words, if the stocking is stopped, the fish will decline gradually. Hence there may be great potential for using silver and bighead carp to counteract the worldwide problem of cyanobacteria blooms in hypereutrophic lakes. The successful practice in Lake Donghu suggested the possibility of this new biomanipulation as an important component of an integrated approach to counteract cyanobacteria blooms, especially in circumstances when nutrients cannot be reduced sufficiently.

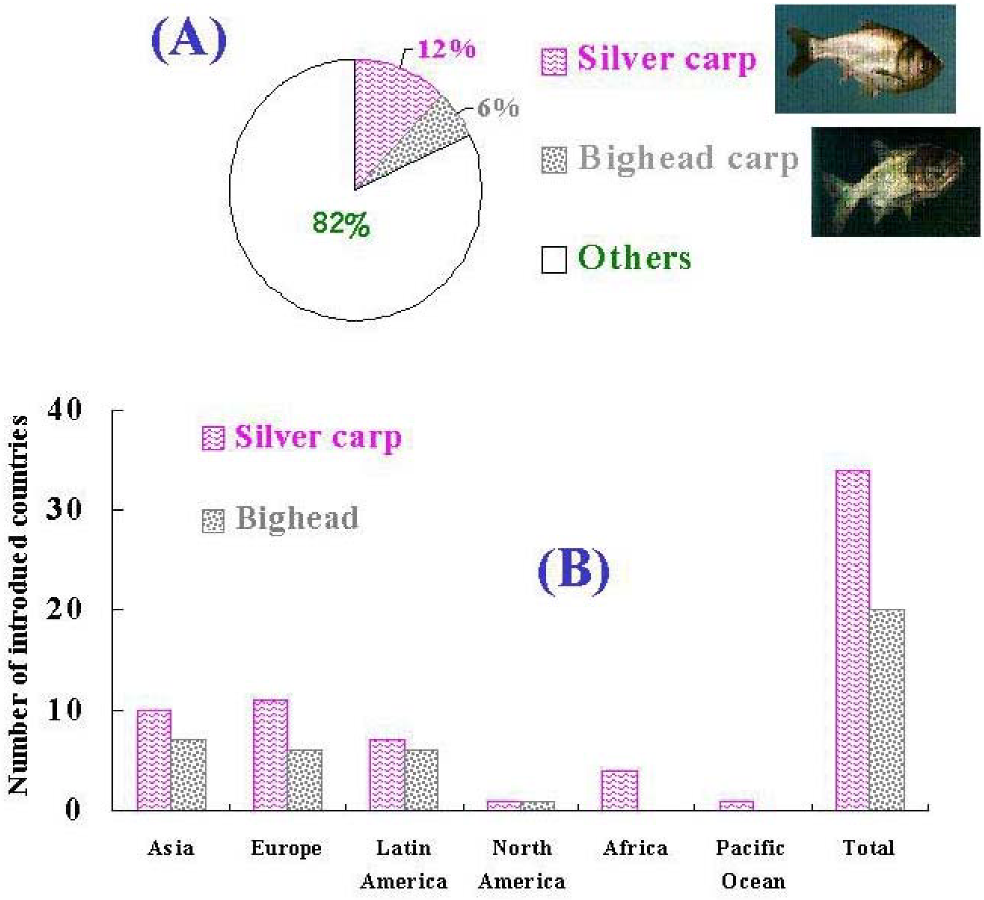

**FIGURE 15.** (A) Proportions of silver carp and bighead in the world's total catch of inland waters in 1989 (data from FAO[49]), and (B) a summary of introduction of silver carp and bighead carp in the world (data from Welcome[50]).

## ACKNOWLEDGMENTS

We wish to give our deep thanks to Dr. K. Havens for his many valuable comments on the manuscript. Prof. Y.F. Shen of the Institute of Hydrobiology, CAS, also gave useful suggestions on this manuscript. Thanks are also due to all the members of the Donghu Experimental Station of Lake Ecosystems for their assistance in carrying out the field work. This work was mainly supported both by the Key Project of CAS (No. KZCX2-403) and the State Key Basic Research and Development Plan (G2000046800). Facilities of the Donghu Experimental Station and the State Key Laboratory for Freshwater Ecology and Biotechnology of China were used in this study.
